# Supplementary material for: Molecular Mechanisms Underlying Qi-Invigorating Effects in Traditional Medicine: Network Pharmacology-Based Study on the Unique Functions of Qi-Invigorating Herb Group
Source: Plants (Basel). 2022 Sep 21;11(19):2470. doi: 10.3390/plants11192470 (PMC9573487; doi:10.3390/plants11192470)
Supplement: Supplementary file 1 [file plants-11-02470-s001.zip › plants-1921797-supplementary.pdf]

Table S1. List of Qi-invigorating herb in Chinese, Korean, English textbooks.

No.	Book name	Herb name	
		**Pharmaceutical name**	**Pinyin name**
1	Zhong yao xue	Radix Ginseng	Ren shen
		Radix Panacis Quinquifolii	Xi yang shen
		Radix Codonopsis	Dang shen
		Radix Pseudostellariae	Tai zi shen
		Radix Astragali	Huang qi
		Rhizoma Atractylodis Macrocephalae	Bai zhu
		Rhizoma Dioscoreae	Shan yao
		Semen Lablab Album	Bian dou
		Radix Glycyrrhizae	Gan cao
		Fructus Jujube	Da zao
		Radix et Caulis Acanthopanacis Senticosi	Ci wu jia
		Rhizoma seu Herba Gynostemmatis	Jiao gu lan
		Radix Rhodiolae Crenulate	Hong jing tian
		Frutus Hippophae	Sha ji
		Saccharum Granorum	Yi tang
		Mel	Feng mi
2	Boncho-hak	Radix Ginseng	Ren shen
		Radix Codonopsis	Dang shen
		Radix Astragali	Huang qi
		Rhizoma Atractylodis Macrocephalae	Bai zhu
		Semen Lablab Album	Bian dou
		Rhizoma Dioscoreae	Shan yao
		Fructus Jujube	Da zao
		Radix Glycyrrhizae	Gan cao
		Mel	Feng mi
3	Chinese medical herbology and pharmacology	Radix Ginseng	Ren shen
		Radix Panacis Quinquifolii	Xi yang shen
		Radix Codonopsis	Dang shen
		Radix Pseudostellariae	Tai zi shen
		Radix Ginseng Japonica	Dong yang shen
		Radix Astragali	Huang qi
		Rhizoma seu Herba Gynostemmatis	Jiao gu lan
		Rhizoma Atractylodis Macrocephalae	Bai zhu
		Rhizoma Dioscoreae	Shan yao
		Semen Lablab Album	Bian dou
		Radix et Caulis Acanthopanacis Senticosi	Ci wu jia

		Radix Glycyrrhizae	Gan cao
		Fructus Jujube	Da zao
		Semen Oryzae	Geng mi
		Mel	Feng mi
		Saccharum Granorum	Yi tang

Table S2. The information of all compounds belongs to Qi-invigorating herbs.

Mol ID	Molecule Name	Herb name
MOL000029	beta-Humulene	Ren shen
MOL000035	beta-Selinene	Ren shen
MOL000036	beta-caryophyllene	Ren shen
MOL000066	alloaromadedrene	Ren shen
MOL000069	palmitic acid	Ren shen
MOL000269	Elemicin	Ren shen
MOL000358	beta-sitosterol	Ren shen
MOL000422	kaempferol	Ren shen
MOL000449	Stigmasterol	Ren shen
MOL000628	darutoside	Ren shen
MOL000676	DBP	Ren shen
MOL000749	Linoleic	Ren shen
MOL000787	Fumarine	Ren shen
MOL000864	MYS	Ren shen
MOL000874	paeonol	Ren shen
MOL000879	methyl palmitate	Ren shen
MOL000886	tetradecane	Ren shen
MOL000908	beta-elemene	Ren shen
MOL000935	Hepanal	Ren shen
MOL000942	(1R,4S,4aR,8aR)-4-isopropyl-1,6-dimethyl-3,4,4a,7,8,8a-hexahydro-2H-naphthalen-1-ol	Ren shen
MOL000968	beta-Bisabolene	Ren shen
MOL001212	Loxanol V	Ren shen
MOL001218	Pisol	Ren shen
MOL001312	9-HEXADECENOIC ACID	Ren shen
MOL001392	Methyl myristate	Ren shen
MOL001396	PENTADECYLIC ACID	Ren shen
MOL001641	METHYL LINOLEATE	Ren shen
MOL001706	2,6-dimethyl-3,7-octadiene-2,6-diol	Ren shen
MOL001738	MLI	Ren shen
MOL001817	Methyl stearate	Ren shen

MOL001818	Methyl palmitelaidate	Ren shen
MOL001819	METHYL PENTADECANOATE	Ren shen
MOL001949	panaxynol	Ren shen
MOL001965	Dauricine (8CI)	Ren shen
MOL002121	(1S,4E,8E,10R)-4,8,11,11-tetramethylbicyclo[8.1.0]undeca-4,8-diene	Ren shen
MOL002136	neocnidilide	Ren shen
MOL002137	OCT	Ren shen
MOL002307	20-Hexadecanoylingenol	Ren shen
MOL002312	[(3S,4R,5R)-5-[[(2R,3S,4S,5R,6S)-6-(2-acetyl-5-methoxyphenoxy)-3,4,5-trihydroxyoxan-2-yl]methoxy]-3,4-dihydroxyoxolan-3-yl]methyl 3,4,5-trihydroxybenzoate	Ren shen
MOL002323	L-Adenosine	Ren shen
MOL002377	Kaempferol-3-arabofuranoside	Ren shen
MOL002526	3691-11-0	Ren shen
MOL002669	Campesteryl ferulate	Ren shen
MOL002879	Diop	Ren shen
MOL003346	Psuedohypericin	Ren shen
MOL003648	Inermin	Ren shen
MOL003845	Folinic acid	Ren shen
MOL003902	methyl (Z)-icos-11-enoate	Ren shen
MOL004100	N-Salicylidene-salicylamine	Ren shen
MOL004174	epsilon-Cadinene	Ren shen
MOL004237	δ-elemene	Ren shen
MOL004275	(1R,4E,7E,11R)-1,5,9,9-tetramethyl-12-oxabicyclo[9.1.0]dodeca-4,7-diene	Ren shen
MOL004492	Chrysanthemaxanthin	Ren shen
MOL004498	12-O-Nicotinoylisolineolone	Ren shen
MOL004647	TDA	Ren shen
MOL005155	ginsenoside Ro_qt	Ren shen
MOL005269	(+)-Maalioxide	Ren shen
MOL005270	n-Heptadecanol	Ren shen
MOL005271	1-HEXADECYNE	Ren shen
MOL005272	13-Tetradecenyl acetate	Ren shen
MOL005273	16-Oxoseratenediol	Ren shen
MOL005274	Neohexane	Ren shen
MOL005275	2,3,4-Trimethyldecane	Ren shen
MOL005276	2,3,8-Trimethyldecane	Ren shen
MOL005277	2,6,10,15-tetramethylheptadecane	Ren shen
MOL005278	2-METHYLTRIDECANE	Ren shen

MOL005279	ginsenoside-Rh1	Ren shen
MOL005280	ginsenoside-Rh1_qt	Ren shen
MOL005281	20(S)-Ginsenoside-Rh1	Ren shen
MOL005282	20(S)-Ginsenoside-Rh1_qt	Ren shen
MOL005283	20(S)-ginsenoside-Rg2	Ren shen
MOL005284	(3S,5R,6S,8R,9R,10R,12R,13R,14R,17S)-17-[(2S)-2-hydroxy-6-methylhept-5-en-2-yl]-4,4,8,10,14-pentamethyl-2,3,5,6,7,9,11,12,13,15,16,17-dodecahydro-1H-cyclopenta[a]phenanthrene-3,6,12-triol	Ren shen
MOL005285	20(s)-protopanaxadiol	Ren shen
MOL005286	20(R)-ginsenoside Rg2	Ren shen
MOL005287	20-(S)-Ginsenoside-Rg3	Ren shen
MOL005288	20-(S)-Ginsenoside-Rg3_qt	Ren shen
MOL005289	3,4-Dimethylheptane	Ren shen
MOL005290	3,5-Dimethyl-p-anisic acid	Ren shen
MOL005291	3-O-beta-D-Glucuronopyranosyl gypsogenin	Ren shen
MOL005292	3-O-beta-D-Glucuronopyranosyl gypsogenin_qt	Ren shen
MOL005293	3-Ethyl-3-methylheptane	Ren shen
MOL005294	3-methylheptane	Ren shen
MOL005295	3-methylundecane	Ren shen
MOL005296	4-Methyldodecane	Ren shen
MOL005297	5-Isobutylnonane	Ren shen
MOL005298	5-heptadec-12-enylresorcinol	Ren shen
MOL005299	5-methyl-tetradecane	Ren shen
MOL005300	6'-Malonylginsenoside Rd1	Ren shen
MOL005301	6'-Malonylginsenoside Rd1_qt1	Ren shen
MOL005302	7-(beta-Xylosyl)cephalomannine	Ren shen
MOL005303	7-Tetradecyne	Ren shen
MOL005304	7alpha-L-Rhamnosyl-6-methoxylutcolin	Ren shen
MOL005305	Nepetin	Ren shen
MOL005306	Acetal	Ren shen
MOL005307	Adenosine triphosphate	Ren shen
MOL005308	Aposiopolamine	Ren shen
MOL005309	Araloside A	Ren shen
MOL005310	(4aS,6aR,6aS,6bR,8aR,10S,12aR,14bR)-10-hydroxy-2,2,6a,6b,9,9,12a-heptamethyl-1,3,4,5,6,6a,7,8,8a,10,11,12,13,14b-tetradecahydropicene-4a-carboxylic acid	Ren shen
MOL005311	Argininyl-fructosyl-glucose	Ren shen
MOL005312	Argininyl-fructosyl-glucose_qt	Ren shen

MOL005313	5-[(3aS,6R,6aR)-2-keto-1,3,3a,4,6,6a-hexahydrothieno[3,4-d]imidazol-6-yl]valeric acid	Ren shen
MOL005314	Celabenzine	Ren shen
MOL005315	(R)-()-Citronellal	Ren shen
MOL005316	MAV	Ren shen
MOL005317	Deoxyharringtonine	Ren shen
MOL005318	Dianthramine	Ren shen
MOL005319	Ditertbutyl phthalate	Ren shen
MOL005320	arachidonate	Ren shen
MOL005321	Frutinone A	Ren shen
MOL005322	Gamma-Selinene	Ren shen
MOL005323	ginsenoside La	Ren shen
MOL005324	ginsenoside La_qt	Ren shen
MOL005325	ginsenoside Ro	Ren shen
MOL005326	Ginsenoside-Ra0	Ren shen
MOL005327	Gypnoside V_qt	Ren shen
MOL005328	Ginsenoside-Ra1	Ren shen
MOL005329	Ginsenoside-Ra2	Ren shen
MOL005330	Ginsenoside-Ra3	Ren shen
MOL005331	ginsenoside Rb1	Ren shen
MOL005332	(3R,5R,8R,9R,10R,12R,13R,14R,17S)-17-[(2S)-2-hydroxy-6-methylhept-5-en-2-yl]-4,4,8,10,14-pentamethyl-2,3,5,6,7,9,11,12,13,15,16,17-dodecahydro-1H-cyclopenta[a]phenanthrene-3,12-diol	Ren shen
MOL005333	ginsenoside-Rb2	Ren shen
MOL005334	(3S,5R,8R,9R,10R,12R,13R,14R,17S)-17-[(2S)-2-hydroxy-6-methylhept-5-en-2-yl]-4,4,8,10,14-pentamethyl-2,3,5,6,7,9,11,12,13,15,16,17-dodecahydro-1H-cyclopenta[a]phenanthrene-3,12-diol	Ren shen
MOL005335	Gypenoside LXIX	Ren shen
MOL005336	ginsenoside-Rc	Ren shen
MOL005337	(2S,3R,4S,5S,6R)-2-[(2S)-2-[(3S,5R,8R,9R,10R,12R,13R,14R,17S)-3-[(2R,3R,4S,5S,6R)-4,5-dihydroxy-6-(hydroxymethyl)-3-[(2S,3R,4S,5S,6R)-3,4,5-trihydroxy-6-(hydroxymethyl)oxan-2-yl]oxyoxan-2-yl]oxy-12-hydroxy-4,4,8,10,14-pentamethyl-2,3,5,6,7,9,11,12,13,15,1	Ren shen
MOL005338	Ginsenoside Re	Ren shen

MOL005340	(3S,5R,6S,8R,9R,10R,12R,13R,14R,17S)-17-[(2R)-2-hydroxy-6-methylhept-5-en-2-yl]-4,4,8,10,14-pentamethyl-2,3,5,6,7,9,11,12,13,15,16,17-dodecahydro-1H-cyclopenta[a]phenanthrene-3,6,12-triol	Ren shen
MOL005341	Sanchinoside C1	Ren shen
MOL005342	Ginsenoside-Rg3	Ren shen
MOL005343	Ginsenoside-Rg3_qt	Ren shen
MOL005344	ginsenoside rh2	Ren shen
MOL005345	(2R,3S,4S,5R,6R)-2-(hydroxymethyl)-6-[[(3S,5R,8R,9R,10R,12R,13R,14R,17S)-12-hydroxy-4,4,8,10,14-pentamethyl-17-[(2Z)-6-methylhepta-2,5-dien-2-yl]-2,3,5,6,7,9,11,12,13,15,16,17-dodecahydro-1H-cyclopenta[a]phenanthren-3-yl]oxy]oxane-3,4,5-triol	Ren shen
MOL005346	Ginsenoside-Rh3_qt	Ren shen
MOL005347	Ginsenoside-Rh4	Ren shen
MOL005348	Ginsenoside-Rh4_qt	Ren shen
MOL005349	Ginsenoside-Rs1	Ren shen
MOL005350	Ginsenoside-Rs2	Ren shen
MOL005351	Ginsenoyne A	Ren shen
MOL005352	Ginsenoyne B	Ren shen
MOL005353	Ginsenoyne C	Ren shen
MOL005354	Ginsenoyne D	Ren shen
MOL005355	Ginsenoyne E	Ren shen
MOL005356	Girinimbin	Ren shen
MOL005357	Gomisin B	Ren shen
MOL005358	L-erythro-isocitric acid	Ren shen
MOL005359	D-erythro-Isocitric acid	Ren shen
MOL005360	malkangunin	Ren shen
MOL005361	Malonylginsenoside Rc	Ren shen
MOL005362	Malonylginsenoside Rc_qt1	Ren shen
MOL005363	Malonylginsenoside Rd	Ren shen
MOL005364	Malonylginsenoside Rd_qt	Ren shen
MOL005365	MAL	Ren shen
MOL005366	Malvic acid	Ren shen
MOL005367	GUP	Ren shen
MOL005368	Methyl tricosanoate	Ren shen
MOL005369	Mycosinol	Ren shen
MOL005370	NN-Dimethyldecanamide	Ren shen
MOL005371	Nonacosanediol-6,8	Ren shen
MOL005372	notoginsenoside R2	Ren shen

MOL005373	notoginsenoside R2_qt	Ren shen
MOL005374	Notoginsenoside R6	Ren shen
MOL005375	Stearyl acetate	Ren shen
MOL005376	Panaxadiol	Ren shen
MOL005378	Panaxytriol	Ren shen
MOL005379	Pancratistatin	Ren shen
MOL005380	Pandamine	Ren shen
MOL005381	2-Formylpyrrole	Ren shen
MOL005382	Ramalic acid	Ren shen
MOL005383	Methylselenocysteine	Ren shen
MOL005384	suchilactone	Ren shen
MOL005385	Suffruticoside A_qt1	Ren shen
MOL005386	Vulgarin	Ren shen
MOL005388	Undecane, 3,6-dimethyl	Ren shen
MOL005389	PANGAMIC ACID	Ren shen
MOL005390	3-[[(2S)-2,4-dihydroxy-3,3-dimethylbutanoyl]amino]propanoic acid	Ren shen
MOL005391	(Z,Z)-alpha-farnesene	Ren shen
MOL005392	alpha-Guttiferin	Ren shen
MOL005394	(Z)-2-methyl-5-[(1S,2R,4R)-2-methyl-3-methylene-2-norbornanyl]pent-2-en-1-ol	Ren shen
MOL005396	cis-Widdrol alpha-epoxide	Ren shen
MOL005397	Dammarane	Ren shen
MOL005398	alexandrin	Ren shen
MOL005399	alexandrin_qt	Ren shen
MOL005400	ginsenoside Rg5	Ren shen
MOL005401	ginsenoside Rg5_qt	Ren shen
MOL005402	Methyl margarate	Ren shen
MOL005403	oleanane	Ren shen
MOL005404	p-Glucosyloxymandelonitrile	Ren shen
MOL006651	Trifolirhizin	Ren shen
MOL007500	panaxatriol	Ren shen
MOL011400	ginsenoside rf	Ren shen
MOL000006	luteolin	Dang shen
MOL000008	apigenin	Dang shen
MOL000009	luteolin-7-o-glucoside	Dang shen
MOL000018	(+/-)-Isoborneol	Dang shen
MOL000027	alpha-Curcumene	Dang shen
MOL000044	atractylenolideII	Dang shen
MOL000045	atractylenolide iii	Dang shen

MOL000069	palmitic acid	Dang shen
MOL000095	delta 7-stigmastenol	Dang shen
MOL000125	(-)-alpha-Pinene	Dang shen
MOL000131	EIC	Dang shen
MOL000261	Myristicin	Dang shen
MOL000303	caprylic acid	Dang shen
MOL000305	lauric acid	Dang shen
MOL000347	Syrigin	Dang shen
MOL000365	syringaresinol	Dang shen
MOL000394	choline	Dang shen
MOL000421	nicotinic acid	Dang shen
MOL000449	Stigmasterol	Dang shen
MOL000508	Friedelin	Dang shen
MOL000617	(14S)-14-methylpalmitic acid	Dang shen
MOL000628	darutoside	Dang shen
MOL000667	1-hexanol	Dang shen
MOL000721	Nonadienal	Dang shen
MOL000748	HMF	Dang shen
MOL000835	EA-fructofuranoside	Dang shen
MOL000860	stearic acid	Dang shen
MOL000867	Heptadekan	Dang shen
MOL000869	Henicosane	Dang shen
MOL000879	methyl palmitate	Dang shen
MOL000885	Dodekan	Dang shen
MOL000890	(+)-alpha-Curcumene	Dang shen
MOL000899	Furanodiene	Dang shen
MOL000905	()-beta-Pinene	Dang shen
MOL000998	2-(3,4-dihydroxyphenyl)-5,7-dihydroxy-3-[(2R,3R,4S,5S)-3,4,5-trihydroxytetrahydropyran-2-yl]oxy-chromone	Dang shen
MOL001006	poriferasta-7,22E-dien-3beta-ol	Dang shen
MOL001160	2-methoxyfuranodiene	Dang shen
MOL001309	6-methylolpyridin-3-ol	Dang shen
MOL001314	Azelex	Dang shen
MOL001392	Methyl myristate	Dang shen
MOL001393	myristic acid	Dang shen
MOL001394	Oktadekan	Dang shen
MOL001396	PENTADECYLIC ACID	Dang shen
MOL001399	TWT	Dang shen
MOL001619	UPL	Dang shen
MOL001620	Pentadecene	Dang shen

MOL001641	METHYL LINOLEATE	Dang shen
MOL001644	Dodecanal	Dang shen
MOL001817	Methyl stearate	Dang shen
MOL001819	METHYL PENTADECANOATE	Dang shen
MOL001887	SRT	Dang shen
MOL002046	hexanoic acid	Dang shen
MOL002140	Perlolyrine	Dang shen
MOL002307	20-Hexadecanoylingenol	Dang shen
MOL002521	beta-Curcumene	Dang shen
MOL002526	3691-11-0	Dang shen
MOL002579	capsaicin	Dang shen
MOL002879	Diop	Dang shen
MOL002943	BuOH	Dang shen
MOL003035	stigmasterol-β-glucoside	Dang shen
MOL003036	ZINC03978781	Dang shen
MOL003050	nonanoic acid	Dang shen
MOL003177	Syringaldehyde	Dang shen
MOL003304	Hentriacontan	Dang shen
MOL003487	D-Friedoolean-14-en-3-one	Dang shen
MOL003509	Nonanol	Dang shen
MOL003766	Shekanin	Dang shen
MOL003767	tectorigenin	Dang shen
MOL003895	5-Methoxymethyl furfural	Dang shen
MOL003896	7-Methoxy-2-methyl isoflavone	Dang shen
MOL004355	Spinasterol	Dang shen
MOL004492	Chrysanthemaxanthin	Dang shen
MOL004498	12-O-Nicotinoylisolineolone	Dang shen
MOL004582	Methyl naphthalene	Dang shen
MOL004623	Encecalin	Dang shen
MOL004652	(2R,3R,4S,5S,6R)-2-[[(3S,5S,9R,10S,13R,14R,17R)-17-[(E,2R,5S)-5-ethyl-6-methylhept-3-en-2-yl]-10,13-dimethyl-2,3,4,5,6,9,11,12,14,15,16,17-dodecahydro-1H-cyclopenta[a]phenanthren-3-yl]oxy]-6-(hydroxymethyl)oxane-3,4,5-triol	Dang shen
MOL004664	heptanoic acid	Dang shen
MOL005270	n-Heptadecanol	Dang shen
MOL005302	7-(beta-Xylosyl)cephalomannine	Dang shen
MOL005304	7alpha-L-Rhamnosyl-6-methoxylutcolin	Dang shen
MOL005321	Frutinone A	Dang shen
MOL005482	FOA	Dang shen

MOL006322	Friedoolean-14-en-3-yl acetate	Dang shen
MOL006554	Taraxerol	Dang shen
MOL006774	stigmast-7-enol	Dang shen
MOL006844	Norharman	Dang shen
MOL006988	NSC405997	Dang shen
MOL007059	3-beta-Hydroxymethyllenetanshiquinone	Dang shen
MOL007514	methyl icosa-11,14-dienoate	Dang shen
MOL008142	Ricinin	Dang shen
MOL008284	BUA	Dang shen
MOL008375	(1R)-2,3,4,9-tetrahydro-1H-$b-carboline-1-carboxylic acid	Dang shen
MOL008376	13-Methyl pentadecanoic acid	Dang shen
MOL008377	Galuteolin	Dang shen
MOL008378	o-(o-Methoxyphenoxy)phenol	Dang shen
MOL008379	tangshenoside I	Dang shen
MOL008380	tangshenoside I_qt	Dang shen
MOL008381	tangshenoside III	Dang shen
MOL008382	tangshenoside III_qt	Dang shen
MOL008383	tangshenoside IV	Dang shen
MOL008384	tangshenoside IV_qt	Dang shen
MOL008385	T-BUTYLBENZENE	Dang shen
MOL008386	2,6-NONADIENOL	Dang shen
MOL008387	3-METHYLCARBAZOLE	Dang shen
MOL008388	L-Sulforaphane	Dang shen
MOL008389	4-Phenylbicyclo[2,2,2]octan-1-ol	Dang shen
MOL008390	5-Mpe-bis(hobz)phenol	Dang shen
MOL008391	5alpha-Stigmastan-3,6-dione	Dang shen
MOL008392	6,6'-Dimethoxygossypol	Dang shen
MOL008393	7-(beta-Xylosyl)cephalomannine_qt	Dang shen
MOL008394	Butylcyclohexane	Dang shen
MOL008395	Codonopsine	Dang shen
MOL008396	Coelogin	Dang shen
MOL008397	Daturilin	Dang shen
MOL008398	Ethyl-p-digallate	Dang shen
MOL008399	fritillaziebinol	Dang shen
MOL008400	glycitein	Dang shen
MOL008401	Henicosanoic acid	Dang shen
MOL008402	BHG	Dang shen
MOL008403	Hexyl-beta-D-glucopyranosyl-(1-2)-beta-D-glucopyranoside	Dang shen
MOL008404	(2S)-2-ammonio-4-[(R)-methylsulfinyl]butyrate	Dang shen

MOL008405	1-Peroxyferolide	Dang shen
MOL008406	Spinoside A	Dang shen
MOL008407	(8S,9S,10R,13R,14S,17R)-17-[(E,2R,5S)-5-ethyl-6-methylhept-3-en-2-yl]-10,13-dimethyl-1,2,4,7,8,9,11,12,14,15,16,17-dodecahydrocyclopenta[a]phenanthren-3-one	Dang shen
MOL008408	Stigmasteryl ferulate	Dang shen
MOL008409	Tangshenoside II	Dang shen
MOL008410	Tangshenoside II_qt	Dang shen
MOL008411	11-Hydroxyrankinidine	Dang shen
MOL008412	alpha-Stigmasta-7,22-dien-3-one	Dang shen
MOL008413	Codopiloic acid	Dang shen
MOL008414	delta22-Stigmasterol	Dang shen
MOL008415	delta7-Stigmastenone-3	Dang shen
MOL008416	delta7-stigmastenol-belta-D-glucopyranoside	Dang shen
MOL008417	ethyl-β-D-fructofuranoside	Dang shen
MOL000033	(3S,8S,9S,10R,13R,14S,17R)-10,13-dimethyl-17-[(2R,5S)-5-propan-2-yloctan-2-yl]-2,3,4,7,8,9,11,12,14,15,16,17-dodecahydro-1H-cyclopenta[a]phenanthren-3-ol	Huang qi
MOL000054	L-	Huang qi
MOL000061	Prolinum	Huang qi
MOL000069	palmitic acid	Huang qi
MOL000098	quercetin	Huang qi
MOL000114	vanillic acid	Huang qi
MOL000131	EIC	Huang qi
MOL000211	Mairin	Huang qi
MOL000239	Jaranol	Huang qi
MOL000251	Rhamnocitrin	Huang qi
MOL000295	alexandrin	Huang qi
MOL000296	hederagenin	Huang qi
MOL000354	isorhamnetin	Huang qi
MOL000356	lupeol	Huang qi
MOL000371	3,9-di-O-methylnissolin	Huang qi
MOL000372	3-Hydroxy-2-picoline	Huang qi
MOL000373	(2S)-4-methoxy-7-methyl-2-[1-methyl-1-[(2S,3R,4S,5S,6R)-3,4,5-trihydroxy-6-methylol-tetrahydropyran-2-yl]oxy-ethyl]-2,3-dihydrofuro[3,2-g]chromen-5-one	Huang qi
MOL000374	5'-hydroxyiso-muronulatol-2',5'-di-O-glucoside	Huang qi
MOL000375	5'-hydroxyiso-muronulatol-2',5'-di-O-glucoside_qt	Huang qi

MOL000376	7,2'-dihydroxy-3',4'-dimethoxyisoflavone-7-O-β-D-glucoside	Huang qi
MOL000377	7-hydroxy-3-(2-hydroxy-3,4-dimethoxy-phenyl)chromone	Huang qi
MOL000378	7-O-methylisomucronulatol	Huang qi
MOL000379	9,10-dimethoxypterocarpan-3-O-β-D-glucoside	Huang qi
MOL000380	(6aR,11aR)-9,10-dimethoxy-6a,11a-dihydro-6H-benzofurano[3,2-c]chromen-3-ol	Huang qi
MOL000381	13-hydroxy-9,11-octadecadienoic acid	Huang qi
MOL000382	Arabinose,d	Huang qi
MOL000383	D-Galacturonic acid, homopolymer	Huang qi
MOL000384	DL-Glucuronic acid	Huang qi
MOL000386	Fucopyranose, L-	Huang qi
MOL000387	Bifendate	Huang qi
MOL000388	gamma-aminobutyric acid	Huang qi
MOL000389	FERULIC ACID (CIS)	Huang qi
MOL000390	daidzein	Huang qi
MOL000391	Ononin	Huang qi
MOL000392	formononetin	Huang qi
MOL000393	Soyasaponin I	Huang qi
MOL000394	choline	Huang qi
MOL000395	GGB	Huang qi
MOL000396	(+)-Syringaresinol	Huang qi
MOL000397	cis-p-Coumarate	Huang qi
MOL000398	isoflavanone	Huang qi
MOL000399	Docosanoate	Huang qi
MOL000400	Flavaxin	Huang qi
MOL000401	astragalosideI	Huang qi
MOL000402	astragalosideI_qt	Huang qi
MOL000403	astragalosideII	Huang qi
MOL000404	astragalosideII_qt	Huang qi
MOL000405	astragalosideⅢ	Huang qi
MOL000406	astragalosideⅢ_qt	Huang qi
MOL000407	astragalosideⅣ	Huang qi
MOL000408	astragalosideⅣ_qt	Huang qi
MOL000409	AstragalosideIV	Huang qi
MOL000410	AstragalosideIV_qt	Huang qi
MOL000411	Astraisoflavanin	Huang qi
MOL000412	Mucronulatol	Huang qi
MOL000413	astrachrysoside A	Huang qi
MOL000414	Caffeate	Huang qi

MOL000415	rutin	Huang qi
MOL000416	Lariciresinol	Huang qi
MOL000417	Calycosin	Huang qi
MOL000418	3'-Hydroxy-4'-methoxyisoflavone-7-O-beta-D-glucoside	Huang qi
MOL000419	astrasieversianin XV	Huang qi
MOL000420	XLS	Huang qi
MOL000421	nicotinic acid	Huang qi
MOL000422	kaempferol	Huang qi
MOL000423	rhamnocitrin-3-O-glucoside	Huang qi
MOL000424	RAM	Huang qi
MOL000425	asernestioside A	Huang qi
MOL000426	asernestioside A_qt	Huang qi
MOL000427	asernestioside B	Huang qi
MOL000428	asernestioside B_qt	Huang qi
MOL000429	Crystal VI	Huang qi
MOL000430	betaine	Huang qi
MOL000431	coumarin	Huang qi
MOL000432	linolenic acid	Huang qi
MOL000433	FA	Huang qi
MOL000434	acetylastragaloside I	Huang qi
MOL000435	acetylastragaloside I_qt	Huang qi
MOL000436	(Z)-1-(2,4-dihydroxyphenyl)-3-(4-hydroxyphenyl)prop-2-en-1-one	Huang qi
MOL000437	Hirsutrin	Huang qi
MOL000438	(3R)-3-(2-hydroxy-3,4-dimethoxyphenyl)chroman-7-ol	Huang qi
MOL000439	isomucronulatol-7,2'-di-O-glucosiole	Huang qi
MOL000440	isomucronulatol-7,2'-di-O-glucosiole_qt	Huang qi
MOL000441	LUPENONE	Huang qi
MOL000442	1,7-Dihydroxy-3,9-dimethoxy pterocarpene	Huang qi
MOL001955	Heriguard	Huang qi
MOL005928	isoferulic acid	Huang qi
MOL000018	(+/-)-Isoborneol	Bai zhu
MOL000019	D-Camphene	Bai zhu
MOL000020	12-senecioyl-2E,8E,10E-atractylentriol	Bai zhu
MOL000021	14-acetyl-12-senecioyl-2E,8E,10E-atractylentriol	Bai zhu
MOL000022	14-acetyl-12-senecioyl-2E,8Z,10E-atractylentriol	Bai zhu
MOL000023	Hemo-sol	Bai zhu
MOL000024	alpha-humulene	Bai zhu
MOL000025	α-Longipinene	Bai zhu
MOL000026	stigmast-22E-en-3beta-ol	Bai zhu

MOL000027	alpha-Curcumene	Bai zhu
MOL000028	α-Amyrin	Bai zhu
MOL000029	beta-Humulene	Bai zhu
MOL000030	(1R)-2-methyl-1-phenylprop-2-en-1-ol	Bai zhu
MOL000031	(3S)-3-[(1R)-1,5-dimethylhex-4-enyl]-6-methylenecyclohexene	Bai zhu
MOL000032	beta-Eudesmol	Bai zhu
MOL000033	(3S,8S,9S,10R,13R,14S,17R)-10,13-dimethyl-17-[(2R,5S)-5-propan-2-yloctan-2-yl]-2,3,4,7,8,9,11,12,14,15,16,17-dodecahydro-1H-cyclopenta[a]phenanthren-3-ol	Bai zhu
MOL000034	2-[(1R,3S,4S)-3-isopropenyl-4-methyl-4-vinylcyclohexyl]propan-2-ol	Bai zhu
MOL000035	beta-Selinene	Bai zhu
MOL000036	beta-caryophyllene	Bai zhu
MOL000037	γ-elemene	Bai zhu
MOL000038	Akridin	Bai zhu
MOL000039	(1S,2R,4R)-Neoiso-dihydrocarveol	Bai zhu
MOL000040	Scopoletol	Bai zhu
MOL000041	PHA	Bai zhu
MOL000042	LPG	Bai zhu
MOL000043	atractylenolide i	Bai zhu
MOL000044	atractylenolideII	Bai zhu
MOL000045	atractylenolide iii	Bai zhu
MOL000046	atractylone	Bai zhu
MOL000047	juniper camphor	Bai zhu
MOL000048	(5E,9Z)-3,6,10-trimethyl-4,7,8,11-tetrahydrocyclodeca[b]furan	Bai zhu
MOL000049	3β-acetoxyatractylone	Bai zhu
MOL000050	GLY	Bai zhu
MOL000051	Polymannose	Bai zhu
MOL000052	Gulutamine	Bai zhu
MOL000053	Methose	Bai zhu
MOL000054	L-	Bai zhu
MOL000055	L-Lysin	Bai zhu
MOL000056	DTY	Bai zhu
MOL000057	DIBP	Bai zhu
MOL000058	2-[(2R,5S,6S)-6,10-dimethylspiro[4.5]dec-9-en-2-yl]propan-2-ol	Bai zhu
MOL000059	uridine	Bai zhu
MOL000060	selina-4(14),7(11)-dien-8-one	Bai zhu

MOL000061	Prolinum	Bai zhu
MOL000062	biatractylolide	Bai zhu
MOL000063	ATRACTYLODES MACROCEPHALA	Bai zhu
MOL000064	D-Serin	Bai zhu
MOL000065	ASI	Bai zhu
MOL000066	alloaromadedrene	Bai zhu
MOL000067	L-Valin	Bai zhu
MOL000068	L-Ile	Bai zhu
MOL000069	palmitic acid	Bai zhu
MOL000070	Ethyl pivaloylacetate	Bai zhu
MOL000071	Istidina	Bai zhu
MOL000072	8β-ethoxy atractylenolide Ⅲ	Bai zhu
MOL000012	Arachic acid	Bai bian dou
MOL012901	D-Homoproline	Bai bian dou
MOL001308	oleic acid	Bai bian dou
MOL000131	EIC	Bai bian dou
MOL002773	beta-carotene	Bai bian dou
MOL003795	CPI	Bai bian dou
MOL003870	gynesine	Bai bian dou
MOL000399	Docosanoate	Bai bian dou
MOL000421	nicotinic acid	Bai bian dou
MOL000675	oleic acid	Bai bian dou
MOL000069	palmitic acid	Bai bian dou
MOL000732	Stachyose	Bai bian dou
MOL000841	raffinose	Bai bian dou
MOL000860	stearic acid	Bai bian dou
MOL000041	PHA	Shan yao
MOL000042	LPG	Shan yao
MOL000050	GLY	Shan yao
MOL000052	Gulutamine	Shan yao
MOL000053	Methose	Shan yao
MOL000054	L-	Shan yao
MOL000055	L-Lysin	Shan yao
MOL000056	DTY	Shan yao
MOL000061	Prolinum	Shan yao
MOL000065	ASI	Shan yao
MOL000067	L-Valin	Shan yao
MOL000068	L-Ile	Shan yao
MOL000071	Istidina	Shan yao
MOL000136	Dioscoreside C	Shan yao

MOL000298	ergosterol	Shan yao
MOL000309	denudatin,a	Shan yao
MOL000310	Denudatin B	Shan yao
MOL000322	Kadsurenone	Shan yao
MOL000388	gamma-aminobutyric acid	Shan yao
MOL000394	choline	Shan yao
MOL000449	Stigmasterol	Shan yao
MOL000546	diosgenin	Shan yao
MOL000628	darutoside	Shan yao
MOL000953	CLR	Shan yao
MOL001559	piperlonguminine	Shan yao
MOL001618	Pellitorin	Shan yao
MOL001736	(-)-taxifolin	Shan yao
MOL002442	Cholesteryl ferulate	Shan yao
MOL003955	D-Cystine	Shan yao
MOL003969	L-Serin	Shan yao
MOL003971	Threonin	Shan yao
MOL004668	GLB	Shan yao
MOL005367	GUP	Shan yao
MOL005427	(24S)-belta-Methyl cholest-8(14)-enol	Shan yao
MOL005428	(24S)-belta-Methyl cholestanol	Shan yao
MOL005429	hancinol	Shan yao
MOL005430	hancinone C	Shan yao
MOL005431	Crotepoxide	Shan yao
MOL005432	ABK	Shan yao
MOL005433	(3S,8S,9S,10R,13R,14S,17R)-17-[(2R,5S)-5-ethyl-6-methylhept-6-en-2-yl]-10,13-dimethyl-2,3,4,7,8,9,11,12,14,15,16,17-dodecahydro-1H-cyclopenta[a]phenanthren-3-ol	Shan yao
MOL005434	24-Methylcholest-5-enyl-3belta-O-glucopyranoside	Shan yao
MOL005435	24-Methylcholest-5-enyl-3belta-O-glucopyranoside_qt	Shan yao
MOL005436	24-Methylcholesta-5,7,22-trien-ebelta-ol	Shan yao
MOL005437	(2S,3S,3aR)-3a-allyl-2-(1,3-benzodioxol-5-yl)-5-methoxy-3-methyl-2,3-dihydrobenzofuran-6-one	Shan yao
MOL005438	campesterol	Shan yao
MOL005439	Ostreasterol	Shan yao
MOL005440	Isofucosterol	Shan yao
MOL005441	BGC	Shan yao
MOL005442	LDP	Shan yao
MOL005443	Batatasin I	Shan yao

MOL005444	3-[2-(3-hydroxyphenyl)ethyl]-5-methoxyphenol	Shan yao
MOL005446	Batatasin IV	Shan yao
MOL005447	L-Xyl	Shan yao
MOL005448	Leucinum	Shan yao
MOL005449	h-Met-h	Shan yao
MOL005450	cholestanol	Shan yao
MOL005451	holest-7-enol	Shan yao
MOL005453	phytic acid	Shan yao
MOL005454	(S)-Allantoin	Shan yao
MOL005455	(-)-ABA	Shan yao
MOL005456	Deltoside	Shan yao
MOL005457	Deltoside_qt	Shan yao
MOL005458	Dioscoreside C_qt	Shan yao
MOL005459	Diosgenin-3-O-beta-D-glucopyranoside	Shan yao
MOL005460	ophipogonin D_qt	Shan yao
MOL005461	Doradexanthin	Shan yao
MOL005462	Methylcimicifugoside	Shan yao
MOL005463	Methylcimicifugoside_qt	Shan yao
MOL005464	(3S,4aS,4bS,8R,8aS,10aR)-3-hydroxy-1,1,4a,8-tetramethyl-7-vinyl-3,4,4b,8,8a,9,10,10a-octahydrophenanthrene-2,5-dione	Shan yao
MOL005465	AIDS180907	Shan yao
MOL005466	1,3,6-trihydroxy-8-(3-hydroxy-3-methyl-butyl)-7-methoxy-2-(3-methylbut-2-enyl)xanthone	Shan yao
MOL000042	LPG	Da zao
MOL000050	GLY	Da zao
MOL000053	Methose	Da zao
MOL000061	Prolinum	Da zao
MOL000065	ASI	Da zao
MOL000067	L-Valin	Da zao
MOL000069	palmitic acid	Da zao
MOL000089	catechol	Da zao
MOL000096	(-)-catechin	Da zao
MOL000098	quercetin	Da zao
MOL000211	Mairin	Da zao
MOL000261	Myristicin	Da zao
MOL000263	oleanolic acid	Da zao
MOL000356	lupeol	Da zao
MOL000357	Sitogluside	Da zao
MOL000358	beta-sitosterol	Da zao

MOL000415	rutin	Da zao
MOL000421	nicotinic acid	Da zao
MOL000429	Crystal VI	Da zao
MOL000449	Stigmasterol	Da zao
MOL000492	(+)-catechin	Da zao
MOL000511	ursolic acid	Da zao
MOL000627	Stepholidine	Da zao
MOL000628	darutoside	Da zao
MOL000663	lignoceric acid	Da zao
MOL000675	oleic acid	Da zao
MOL000734	GLO	Da zao
MOL000749	Linoleic	Da zao
MOL000771	p-coumaric acid	Da zao
MOL000783	Protoporphyrin	Da zao
MOL000787	Fumarine	Da zao
MOL000842	sucrose	Da zao
MOL001335	WLN: Q1R	Da zao
MOL001454	berberine	Da zao
MOL001468	MLT	Da zao
MOL001501	Daturic acid	Da zao
MOL001522	(S)-Coclaurine	Da zao
MOL001540	Spinosin	Da zao
MOL001551	Trochol	Da zao
MOL001739	zoomaric acid	Da zao
MOL001841	IES	Da zao
MOL001886	Tar	Da zao
MOL001952	Sinapyl alcohol	Da zao
MOL001996	Betulonic acid	Da zao
MOL002307	20-Hexadecanoylingenol	Da zao
MOL002722	Vomifoliol	Da zao
MOL002730	Vitamin- G	Da zao
MOL002731	(4S)-4-hydroxy-3,5,5-trimethyl-4-[(E,3R)-3-[(2R,3R,4S,5S,6R)-3,4,5-trihydroxy-6-(hydroxymethyl)tetrahydropyran-2-yl]oxybut-1-enyl]cyclohex-2-en-1-one	Da zao
MOL002773	beta-carotene	Da zao
MOL003410	Ziziphin_qt	Da zao
MOL003776	oleanonic acid	Da zao
MOL004082	TGL	Da zao
MOL004349	Ruvoside	Da zao

MOL004350	Ruvoside_qt	Da zao
MOL004498	12-O-Nicotinoylisolineolone	Da zao
MOL004550	Pomolic acid	Da zao
MOL005239	Lysicamine	Da zao
MOL005360	malkangunin	Da zao
MOL005386	Vulgarin	Da zao
MOL005390	3-[[(2S)-2,4-dihydroxy-3,3-dimethylbutanoyl]amino]propanoic acid	Da zao
MOL005448	Leucinum	Da zao
MOL005508	Glucosol	Da zao
MOL005559	Maslinic acid	Da zao
MOL006077	Thiamine	Da zao
MOL006837	spinosin_qt	Da zao
MOL006986	Nornuciferine	Da zao
MOL007213	Nuciferin	Da zao
MOL007341	alphitotic acid	Da zao
MOL008029	N-(2'-hydroxytetraeosanol)-2-amino-8-octadecene-1,3,4-triol	Da zao
MOL008032	(1S,2R,4aS,6aR,6aS,6bR,8aR,12aR,14bS)-1,2,6a,6b,9,9,12a-heptamethyl-10-oxo-1,2,3,4,5,6,6a,7,8,8a,11,12,13,14b-tetradecahydropicene-4a-carboxylic acid	Da zao
MOL008034	21302-79-4	Da zao
MOL008647	Moupinamide	Da zao
MOL011038	(+)-Catechin-5-O-glucoside	Da zao
MOL011039	Vaccenic acid	Da zao
MOL011543	Zizybeoside I	Da zao
MOL012921	stepharine	Da zao
MOL012939	Scutianine C	Da zao
MOL012940	Spiradine A	Da zao
MOL012941	Sylvestrene	Da zao
MOL012942	Tannin	Da zao
MOL012943	Ziziphin	Da zao
MOL012944	Zizyphine A	Da zao
MOL012945	Zizyphus saponin III	Da zao
MOL012946	zizyphus saponin I_qt	Da zao
MOL012947	Zizyphus saponin II	Da zao
MOL012948	Zuztvisude I	Da zao
MOL012949	(4S)-4-hydroxy-4-[(E,3S)-3-hydroxybut-1-enyl]-3,5,5-trimethylcyclohex-2-en-1-one	Da zao
MOL012950	CMP	Da zao

MOL012951	PCG	Da zao
MOL012952	Daechualkaloid A	Da zao
MOL012953	Jubanine-A	Da zao
MOL012954	daechuine S3	Da zao
MOL012955	24-Dehydrocholesterol	Da zao
MOL012956	18658-41-8	Da zao
MOL012957	ADA	Da zao
MOL012958	jujubasaponin IV	Da zao
MOL012959	jujubasaponin IV_qt	Da zao
MOL012960	jujuboside A	Da zao
MOL012961	jujuboside A_qt	Da zao
MOL012962	swertisin	Da zao
MOL012963	zizybeoside II	Da zao
MOL012964	zizyphus saponin I	Da zao
MOL012965	zizyvoside II	Da zao
MOL012966	(4R)-4-hydroxy-4-[(E,3S)-3-hydroxybut-1-enyl]-3,5,5-trimethylcyclohex-2-en-1-one	Da zao
MOL012967	zizyvoside I	Da zao
MOL012968	1-(4-Coumaroyl)alpha-rhamnopyranose	Da zao
MOL012969	2α-hydroxyoleanolic acid	Da zao
MOL012970	2α-hydroxyursolic acid	Da zao
MOL012971	3-O-cis-p-coumaroyl alphitolic acid	Da zao
MOL012972	6'-O-p-Coumaroylgenipingentiobioside	Da zao
MOL012973	Adouetine X	Da zao
MOL012974	Asimilobine	Da zao
MOL012975	Catharanthamine	Da zao
MOL012976	coumestrol	Da zao
MOL012977	Daechuine S10	Da zao
MOL012978	Daechuine S26	Da zao
MOL012979	Daechuine S5	Da zao
MOL012980	Daechuine S6	Da zao
MOL012981	Daechuine S7	Da zao
MOL012982	Daechuine S8-1	Da zao
MOL012983	Jujubasaponin VI	Da zao
MOL012984	Jujubasaponin VI_qt	Da zao
MOL012985	Jujubasaponin V	Da zao
MOL012986	Jujubasaponin V_qt	Da zao
MOL012987	Jujubogenin	Da zao
MOL012988	Jujuboside A1	Da zao
MOL012989	Jujuboside C_qt	Da zao

MOL012990	Jujuboside C	Da zao
MOL012991	Jujuboside	Da zao
MOL012992	Mauritine D	Da zao
MOL012993	Mauritine A	Da zao
MOL012995	zizyberanalic acid	Da zao
MOL013357	(3S,6R,8S,9S,10R,13R,14S,17R)-17-[(1R,4R)-4-ethyl-1,5-dimethylhexyl]-10,13-dimethyl-2,3,6,7,8,9,11,12,14,15,16,17-dodecahydro-1H-cyclopenta[a]phenanthrene-3,6-diol	Da zao
MOL000105	protocatechuic acid	Gan cao
MOL001097	o-xylene	Gan cao
MOL001098	m-xylene	Gan cao
MOL001099	p-xylene	Gan cao
MOL000118	(L)-alpha-Terpineol	Gan cao
MOL000012	Arachic acid	Gan cao
MOL001484	Inermine	Gan cao
MOL001543	Vicenin-2	Gan cao
MOL001599	α-cubebol	Gan cao
MOL001696	Morusin	Gan cao
MOL001737	ICO	Gan cao
MOL001789	isoliquiritigenin	Gan cao
MOL001792	DFV	Gan cao
MOL001850	Izoforon	Gan cao
MOL000211	Mairin	Gan cao
MOL002137	OCT	Gan cao
MOL002166	ISOHEPTANE	Gan cao
MOL002198	Heptan	Gan cao
MOL002311	Glycyrol	Gan cao
MOL000239	Jaranol	Gan cao
MOL002547	21987_FLUKA	Gan cao
MOL002565	Medicarpin	Gan cao
MOL000263	oleanolic acid	Gan cao
MOL002678	EB	Gan cao
MOL002693	nicotiflorin	Gan cao
MOL002844	Pinocembrin	Gan cao
MOL002850	butylated hydroxytoluene	Gan cao
MOL002943	BuOH	Gan cao
MOL003218	Neouralenol	Gan cao
MOL000354	isorhamnetin	Gan cao
MOL000359	sitosterol	Gan cao
MOL003656	Lupiwighteone	Gan cao

MOL003662	7,4'-Dihydroxyflavone	Gan cao
MOL003686	Narcissoside	Gan cao
MOL003896	7-Methoxy-2-methyl isoflavone	Gan cao
MOL000392	formononetin	Gan cao
MOL003985	2-Caren-10-al	Gan cao
MOL000040	Scopoletol	Gan cao
MOL000415	rutin	Gan cao
MOL000417	Calycosin	Gan cao
MOL000422	kaempferol	Gan cao
MOL004328	naringenin	Gan cao
MOL000437	Hirsutrin	Gan cao
MOL000445	8-Prenylwighteone	Gan cao
MOL004589	Methylheptane	Gan cao
MOL000467	Castanin	Gan cao
MOL004723	beta-Terpinene	Gan cao
MOL000475	anethole	Gan cao
MOL004801	2',7-Dihydroxy-4'-methoxyisoflavan-7-O-β-d-glucopyranoside	Gan cao
MOL004802	(E)-1-butoxyhex-2-ene	Gan cao
MOL004803	3-Hydroxyglabrol	Gan cao
MOL004804	18beta-glycyrrhetinic acid	Gan cao
MOL004805	(2S)-2-[4-hydroxy-3-(3-methylbut-2-enyl)phenyl]-8,8-dimethyl-2,3-dihydropyrano[2,3-f]chromen-4-one	Gan cao
MOL004806	euchrenone	Gan cao
MOL004807	glucuronic acid	Gan cao
MOL004808	glyasperin B	Gan cao
MOL004809	glyasperin E	Gan cao
MOL004810	glyasperin F	Gan cao
MOL004811	Glyasperin C	Gan cao
MOL004812	glyasperins D	Gan cao
MOL004813	glyasperins Z	Gan cao
MOL004814	Isotrifoliol	Gan cao
MOL004815	(E)-1-(2,4-dihydroxyphenyl)-3-(2,2-dimethylchromen-6-yl)prop-2-en-1-one	Gan cao
MOL004816	(2R)-1-[2,4-dihydroxy-5-(3-methylbut-2-enyl)phenyl]-2-hydroxy-3-[4-hydroxy-3-(3-methylbut-2-enyl)phenyl]propan-1-one	Gan cao
MOL004817	kanzonols K	Gan cao
MOL004818	kanzonols L	Gan cao
MOL004819	kanzonols T	Gan cao

MOL004820	kanzonols W	Gan cao
MOL004821	kanzonols X	Gan cao
MOL004822	(E)-1-(2,4-dihydroxyphenyl)-3-[4-hydroxy-3-(3-methylbut-2-enyl)phenyl]prop-2-en-1-one	Gan cao
MOL004823	licoagropin	Gan cao
MOL004824	(2S)-6-(2,4-dihydroxyphenyl)-2-(2-hydroxypropan-2-yl)-4-methoxy-2,3-dihydrofuro[3,2-g]chromen-7-one	Gan cao
MOL004825	glyinflanin A	Gan cao
MOL005812	naringin	Gan cao
MOL004827	Semilicoisoflavone B	Gan cao
MOL004828	Glepidotin A	Gan cao
MOL004829	Glepidotin B	Gan cao
MOL004830	Octadiene	Gan cao
MOL004831	(E)-1-[2,4-dihydroxy-3-(3-methylbut-2-enyl)phenyl]-3-[4-hydroxy-3-(3-methylbut-2-enyl)phenyl]prop-2-en-1-one	Gan cao
MOL004832	WLN: 4OVR	Gan cao
MOL004833	Phaseolinisoflavan	Gan cao
MOL004834	3-(2-hydroxy-4-methoxyphenyl)-2H-chromen-7-ol	Gan cao
MOL004835	Glypallichalcone	Gan cao
MOL004836	echinatin	Gan cao
MOL004837	Karenzu DK2	Gan cao
MOL004838	8-(6-hydroxy-2-benzofuranyl)-2,2-dimethyl-5-chromenol	Gan cao
MOL004839	(1S,2S)-1,2-dimethylcyclopentane	Gan cao
MOL004840	Liconeolignan	Gan cao
MOL004841	Licochalcone B	Gan cao
MOL004842	licochalcone C	Gan cao
MOL004843	licochalconeD	Gan cao
MOL004844	glabrol	Gan cao
MOL004845	apioglycyrrhizin	Gan cao
MOL004846	apioglycyrrhizin_qt	Gan cao
MOL004847	2,2-DIMETHYLPENTANE	Gan cao
MOL004848	licochalcone G	Gan cao
MOL004849	3-(2,4-dihydroxyphenyl)-8-(1,1-dimethylprop-2-enyl)-7-hydroxy-5-methoxy-coumarin	Gan cao
MOL004850	liquoric acid	Gan cao
MOL004851	Licoflavone	Gan cao
MOL004852	7-hydroxy-2-[4-hydroxy-3-(3-methylbut-2-enyl)phenyl]-6-(3-methylbut-2-enyl)chromone	Gan cao
MOL004853	Licoflavonol	Gan cao
MOL004385	Yinyanghuo D	Gan cao

MOL004855	Licoricone	Gan cao
MOL004856	Gancaonin A	Gan cao
MOL004857	Gancaonin B	Gan cao
MOL004858	Gancaonin C	Gan cao
MOL004859	2,3-dimethylhexane	Gan cao
MOL000486	Prunetin	Gan cao
MOL004860	licorice glycoside E	Gan cao
MOL004861	Gancaonin D	Gan cao
MOL004862	(2R)-2-[3,4-dihydroxy-5-(3-methylbut-2-enyl)phenyl]-5,7-dihydroxy-8-(3-methylbut-2-enyl)chroman-4-one	Gan cao
MOL004863	3-(3,4-dihydroxyphenyl)-5,7-dihydroxy-8-(3-methylbut-2-enyl)chromone	Gan cao
MOL004864	5,7-dihydroxy-3-(4-methoxyphenyl)-8-(3-methylbut-2-enyl)chromone	Gan cao
MOL004865	5,7-dihydroxy-3-(2-hydroxy-4-methoxy-phenyl)-6-(3-methylbut-2-enyl)chromone	Gan cao
MOL004866	2-(3,4-dihydroxyphenyl)-5,7-dihydroxy-6-(3-methylbut-2-enyl)chromone	Gan cao
MOL004867	Gancaonin P	Gan cao
MOL004868	Gancaonin Q	Gan cao
MOL004869	Gancaonin R	Gan cao
MOL004870	Gancaonin S	Gan cao
MOL004871	(3S)-2,3-dimethylpentane	Gan cao
MOL004872	gancaonin T	Gan cao
MOL004873	Gancaonin U	Gan cao
MOL004874	Gancaonin V	Gan cao
MOL004875	3-[4,6-dihydroxy-2-methoxy-3-(3-methylbut-2-enyl)phenyl]-7-hydroxy-chromone	Gan cao
MOL004876	Glycyram	Gan cao
MOL004877	Licoricidin	Gan cao
MOL004878	Glycycoumarin	Gan cao
MOL004879	Glycyrin	Gan cao
MOL004880	5,6,7,8-Tetrahydro-2,4-dimethylquinoline	Gan cao
MOL004881	(E)-1-[2,4-dihydroxy-3-(3-methylbut-2-enyl)phenyl]-3-(2,4-dihydroxyphenyl)prop-2-en-1-one	Gan cao
MOL004882	Licocoumarone	Gan cao
MOL004883	Licoisoflavone	Gan cao
MOL004884	Licoisoflavone B	Gan cao
MOL004885	licoisoflavanone	Gan cao
MOL004886	licorice-saponin C2	Gan cao

MOL004887	licorice-saponin C2_qt	Gan cao
MOL004888	licorice-saponin F3	Gan cao
MOL004889	licorice-saponin F3_qt	Gan cao
MOL004890	(4S)-2,4-dimethylhexane	Gan cao
MOL004891	shinpterocarpin	Gan cao
MOL004892	licorice-saponin G2	Gan cao
MOL004893	licorice-saponin G2_qt	Gan cao
MOL004894	licorice-saponin H2	Gan cao
MOL004895	licorice-saponin H2_qt	Gan cao
MOL004896	licorice-saponin J2	Gan cao
MOL004897	licorice-saponin J2_qt	Gan cao
MOL004898	(E)-3-[3,4-dihydroxy-5-(3-methylbut-2-enyl)phenyl]-1-(2,4-dihydroxyphenyl)prop-2-en-1-one	Gan cao
MOL004899	licorice-saponin B2	Gan cao
MOL004900	licorice-saponin K2	Gan cao
MOL004901	licorice-saponin K2_qt	Gan cao
MOL004902	glycyrrhetol	Gan cao
MOL004903	liquiritin	Gan cao
MOL004904	licopyranocoumarin	Gan cao
MOL004905	3,22-Dihydroxy-11-oxo-delta(12)-oleanene-27-alpha-methoxycarbonyl-29-oic acid	Gan cao
MOL004906	Hispaglabridin B	Gan cao
MOL004907	Glyzaglabrin	Gan cao
MOL004908	Glabridin	Gan cao
MOL004909	glabrolide	Gan cao
MOL004910	Glabranin	Gan cao
MOL004911	Glabrene	Gan cao
MOL004912	Glabrone	Gan cao
MOL004913	1,3-dihydroxy-9-methoxy-6-benzofurano[3,2-c]chromenone	Gan cao
MOL004914	1,3-dihydroxy-8,9-dimethoxy-6-benzofurano[3,2-c]chromenone	Gan cao
MOL004915	Eurycarpin A	Gan cao
MOL004916	2-methyl-5-propyl -nonane	Gan cao
MOL004917	glycyroside	Gan cao
MOL004918	HEX	Gan cao
MOL004919	Sextone B	Gan cao
MOL004920	Methylcyclopentane	Gan cao
MOL004921	Docosyl caffeate	Gan cao
MOL004922	2-methyl-6-ethyl decane	Gan cao

MOL000391	Ononin	Gan cao
MOL004924	(-)-Medicocarpin	Gan cao
MOL004925	vitexin	Gan cao
MOL004926	4H-1-Benzopyran-4-one, 2-(4-(beta-D-glucopyranosyloxy)phenyl)-2,3-dihydro-5,7-dihydroxy-, (2S)-	Gan cao
MOL004927	Hispaglabridin A	Gan cao
MOL004928	violanthin	Gan cao
MOL004929	Pentadecanol	Gan cao
MOL004930	Uralenol	Gan cao
MOL004931	Uralenol-3-methylether	Gan cao
MOL004932	glycyrrhizin	Gan cao
MOL004933	uralsaponin B	Gan cao
MOL004934	Isohexane	Gan cao
MOL004935	Sigmoidin-B	Gan cao
MOL004936	Uralene	Gan cao
MOL004937	uralenneoside	Gan cao
MOL004938	schaftoside	Gan cao
MOL004939	Nortangeretin	Gan cao
MOL004940	neoliquiritin	Gan cao
MOL004941	(2R)-7-hydroxy-2-(4-hydroxyphenyl)chroman-4-one	Gan cao
MOL004942	(E)-dodec-2-ene	Gan cao
MOL004943	neoisoliquiritin	Gan cao
MOL004944	Cyclobutanol, 1-ethyl-	Gan cao
MOL004945	(2S)-7-hydroxy-2-(4-hydroxyphenyl)-8-(3-methylbut-2-enyl)chroman-4-one	Gan cao
MOL004946	2-Tetradecanone	Gan cao
MOL004947	Isoviolanthin	Gan cao
MOL004948	Isoglycyrol	Gan cao
MOL004949	Isolicoflavonol	Gan cao
MOL004950	isoglycycoumarin	Gan cao
MOL004951	Isoliquiritin	Gan cao
MOL004952	licuraside	Gan cao
MOL004953	Liquiritin apioside	Gan cao
MOL004954	isograbrol	Gan cao
MOL004955	isoglabrolide	Gan cao
MOL004956	Isoononin	Gan cao
MOL004957	HMO	Gan cao
MOL004958	Isoschaftoside	Gan cao
MOL004959	1-Methoxyphaseollidin	Gan cao

MOL004960	22β-acetylglabric acid	Gan cao
MOL004961	Quercetin der.	Gan cao
MOL004962	24-Hydroxy-11-deoxyglycyrrhetic acid	Gan cao
MOL004963	24-Hydroxyglycyrrhetic acid	Gan cao
MOL004964	(Z)-1-(2,4-dihydroxyphenyl)-3-phenylprop-2-en-1-one	Gan cao
MOL004965	3'(γ,γ-dimethylallyl)-kievitone	Gan cao
MOL004966	3'-Hydroxy-4'-O-Methylglabridin	Gan cao
MOL004967	3,3-Dimethylpentane	Gan cao
MOL004968	3,4,3',4'-Tetrahydroxy-2-methoxychalcone	Gan cao
MOL004969	2-Ethyl-p-xylene	Gan cao
MOL000497	licochalcone a	Gan cao
MOL004970	3-methylheptane	Gan cao
MOL004971	3-methylhexane	Gan cao
MOL004972	3-Methylpentane	Gan cao
MOL004973	3-Ethylpentane	Gan cao
MOL004974	3'-Methoxyglabridin	Gan cao
MOL004975	3β-formylglabrolide	Gan cao
MOL004976	Daidzein dimethyl ether	Gan cao
MOL004977	1-Methoxyficifolinol	Gan cao
MOL004978	2-[(3R)-8,8-dimethyl-3,4-dihydro-2H-pyrano[6,5-f]chromen-3-yl]-5-methoxyphenol	Gan cao
MOL004979	4,2',4',alpha-Tetrahydroxydihydrochalcone	Gan cao
MOL004980	Inflacoumarin A	Gan cao
MOL004981	1-(5-hydroxy-2,2-dimethylchromen-6-yl)-3-(4-hydroxyphenyl)prop-2-en-1-one	Gan cao
MOL004982	2,6,10-trimethyl-dodecane	Gan cao
MOL004983	5,6,7,8-Tetrahydro-4-methylquinoline	Gan cao
MOL005015	Licoriisoflavan A	Gan cao
MOL004985	icos-5-enoic acid	Gan cao
MOL004986	6″-O-acetylliquiritin	Gan cao
MOL004987	11-deoxyglycyrrhetic acid	Gan cao
MOL004988	Kanzonol F	Gan cao
MOL004989	6-prenylated eriodictyol	Gan cao
MOL004990	7,2',4'-trihydroxy - 5-methoxy-3 - arylcoumarin	Gan cao
MOL004991	7-Acetoxy-2-methylisoflavone	Gan cao
MOL004992	7-hydroxy-2-methyl-3-phenyl-chromone	Gan cao
MOL004993	8-prenylated eriodictyol	Gan cao
MOL004994	12-methyltetradecanoate	Gan cao
MOL004995	Kanzonol H	Gan cao
MOL004996	gadelaidic acid	Gan cao

MOL004997	Araboglycyrrhizin	Gan cao
MOL004998	Araboglycyrrhizin_qt	Gan cao
MOL004999	Artonin E	Gan cao
MOL000500	Vestitol	Gan cao
MOL005000	Gancaonin G	Gan cao
MOL005001	Gancaonin H	Gan cao
MOL005002	beta-Glycyrrhetinic acid	Gan cao
MOL005003	Licoagrocarpin	Gan cao
MOL005004	Gancaonin I	Gan cao
MOL005005	Glyasperin A	Gan cao
MOL005006	Glyasperins K	Gan cao
MOL005007	Glyasperins M	Gan cao
MOL005008	Glycyrrhiza flavonol A	Gan cao
MOL005009	Corylifolinin	Gan cao
MOL005010	Kanzonol E	Gan cao
MOL005011	Kanzonol Z	Gan cao
MOL005012	Licoagroisoflavone	Gan cao
MOL005013	18α-hydroxyglycyrrhetic acid	Gan cao
MOL005014	Licorice glycoside A	Gan cao
MOL005016	Odoratin	Gan cao
MOL005017	Phaseol	Gan cao
MOL005018	Xambioona	Gan cao
MOL005019	(2R)-7-hydroxy-2-[4-hydroxy-3-(3-methylbut-2-enyl)phenyl]chroman-4-one	Gan cao
MOL005020	dehydroglyasperins C	Gan cao
MOL005021	Mipax	Gan cao
MOL000511	ursolic acid	Gan cao
MOL000561	Astragalin	Gan cao
MOL000057	DIBP	Gan cao
MOL000668	PENTYLFURAN	Gan cao
MOL000671	()-Menthol	Gan cao
MOL000676	DBP	Gan cao
MOL000703	2-heptanone	Gan cao
MOL000705	WLN: VH6	Gan cao
MOL000098	quercetin	Gan cao
